# Agreement between early-phase amyloid-PET and pulsed arterial spin labeling in a memory clinic cohort

**DOI:** 10.1007/s00109-025-02545-w

**Published:** 2025-05-20

**Authors:** F. Ribaldi, A. J. Mendes, I. Boscolo Galazzo, V. Natale, G. Mathoux, M. Pievani, K. O. Lovblad, M. Scheffler, G. B. Frisoni, V. Garibotto, F. B. Pizzini

**Affiliations:** 1 Geneva Memory Center, Department of Rehabilitation and Geriatrics, Geneva University Hospitals, Geneva, Switzerland; 2 Laboratory of Neuroimaging of Aging (LANVIE), University of Geneva, Geneva, Switzerland; 3 Department of Engineering for Innovation Medicine, University of Verona, Verona, Italy; 4 Department of Diagnostic and Public Health, Rivoli Hospital, Rivoli (TO), Italy; 5 Division of Nuclear Medicine and Molecular Imaging, Diagnostic Department, Geneva University Hospitals, Geneva, Switzerland; 6 Laboratory Alzheimer’s Neuroimaging & Epidemiology, IRCCS Istituto Centro San Giovanni Di Dio Fatebenefratelli, Brescia, Italy; 7 Neurodiagnostic and Neurointerventional Division, Diagnostic Department, Geneva University Hospitals, Geneva, Switzerland; 8 Division of Radiology, Geneva University Hospitals, Geneva, Switzerland; 9 Laboratory of Neuroimaging and Innovative Molecular Tracers (NIMTlab), Neurocenter and Faculty of Medicine, Geneva University, University of Geneva, Geneva, Switzerland; 10 CIBM Center for Biomedical Imaging, Geneva, Switzerland; 11 Radiology and Department of Engineering forInnovation Medicine, Verona University, Verona, Italy

**Keywords:** Perfusion, Arterial spin labeling, Early-phase PET, Cerebral blood flow

## Abstract

**Abstract:**

Relative cerebral blood flow (rCBF), assessed using pulsed arterial spin labeling (pASL) MRI, and the standardized uptake value ratio (SUVr) in early-phase amyloid-PET (ePET) are used as proxies for brain perfusion. These methods have the potential to streamline clinical workflows and reduce the burden on patients by eliminating the need for additional procedures. While both techniques have shown good agreement with the gold standard for glucose metabolism assessment, F-fluorodeoxyglucose-PET, a direct comparison between them has yet to be fully clarified. This retrospective study aimed to compare perfusion-like data from pASL (rCBF) and ePET (SUVr) in a memory clinic cohort. We included 46 subjects (69 ± 8 years; 37 women) from the Geneva Memory Center (cognitively impaired-CI *n* = 29; cognitively unimpaired-CU *n* = 17), with available pASL and ePET. We evaluated the association between rCBF and SUVr values across 18 cortical and subcortical regions using linear regression and the within-subject coefficient of variation (wsCV). Regional differences between CU and CI groups were assessed using linear regression model corrected for age. We observed significant association between rCBF and SUVr in precuneus (*β* = 0.69, wsCV = 16.9), angular gyrus (*β* = 0.64, wsCV = 19.4), and hippocampus (*β* = 0.23, wsCV = 16.1). Additionally, significant differences in rCBF between CU and CI were also observed in the posterior cingulate, precuneus, calcarine, hippocampus, and composite (*p* < 0.05), while SUVr showed significant differences only in the hippocampus. Our findings indicate weak to moderate local correlations between the two techniques. However, both exhibited differing regional perfusion levels in CU and CI groups, with rCBF showing more regional differences between cognitive stages in comparison with SUVr.

**Key messages:**

- rCBF is assessed through pASL MRI and SUVr through ePET, both serving as proxies of brain perfusion.
- Weak to moderate associations between rCBF and SUVr were found in a number of brain regions.
- rCBF and SUVr differences between cognitive stages were observed mostly in cortical and subcortical regions respectively.
- Both techniques were able to identify AD perfusion-like differences expected in cognitively impaired vs unimpaired.

**Supplementary Information:**

The online version contains supplementary material available at 10.1007/s00109-025-02545-w.

## Introduction

The main hallmarks of Alzheimer’s disease (AD) include amyloid-beta plaques, neurofibrillary tangles, and neurodegeneration, which can be detected using cerebrospinal fluid analysis, imaging techniques, or, more recently, blood biomarkers. Neurodegeneration is specifically detected by observing patterns of brain hypometabolism on positron emission tomography (PET) scans or medial temporal atrophy (MTA) on magnetic resonance imaging (MRI).

F-fluorodeoxyglucose (FDG)-PET is considered the gold standard for describing patterns of hypometabolism; however, both amyloid-PET and arterial spin labeling (ASL) MRI have the capacity to estimate brain perfusion, serving as indirect indicator of brain metabolism. Amyloid-PET tracers, due to their lipophilic nature, have a high first-pass influx rate (K1) [1] which correlates with regional cerebral blood flow (CBF) because of the high extraction fraction [2]. During amyloid-PET scanning, an early static acquisition can provide perfusion-like data, which has been shown to exhibit good agreement with the gold standard [3, 4]. ASL MRI is a non-invasive alternative that quantifies CBF by magnetically labeling blood before it enters the brain tissue, thereby using it as an intrinsic flow tracer. The reliability of ASL in measuring CBF has been widely investigated and is becoming part of the diagnostic workup for patients with cognitive decline [5, 6]. ASL can be considered a potential non-invasive alternative to FDG-PET in AD, as patterns of ASL hypoperfusion in subjects with mild cognitive impairment (MCI) and AD have been shown to be comparable to FDG-PET hypometabolism [7–9].

Given the potential of both early frames (ePET) and pulsed ASL to provide perfusion information, they could reduce the clinic workup and lessen the burden on patients by eliminating the need for additional procedures, such as FDG-PET. However, it is essential to evaluate the concordance of perfusion information provided by ePET and pulsed ASL. To date, only two studies have assessed their concordance in AD patients, demonstrating hypoperfusion in similar regions [10, 11]. In both cases, the tracer used for PET was [11 C] Pittsburgh compound-B (PIB) [10], and one study focused on subjects at risk of autosomal AD [10]. In the current study, we aimed to assess the comparability of perfusion-like measures obtained from early frames of static amyloid-PET and pulsed ASL in a memory clinic cohort. Additionally, we intended to compare the regional perfusion-like measures between cognitively impaired (CI) and unimpaired (CU) subjects.

## Methods

### Study design and subjects’ selection

Subjects for this study were selected retrospectively from the Geneva Memory Center (GMC) cohort between 2016 and 2021, a naturalistic cohort of individuals with varying degrees of cognitive decline, from subjective to objective, who are under observation at the GMC of the Geneva University Hospitals. GMC subjects undergo a comprehensive diagnostic workup, including clinical and neuropsychological assessments, a neurological exam, and MRI. Thanks to several interconnected research projects, some subjects also undergo amyloid PET scans or other procedures (CSF, blood, Tau PET). Further information is available in [12].

Inclusion criteria for this retrospective study required the availability of both relative CBF (rCBF) assessed through pulsed ASL and standardized uptake value ratio (SUVr) in early frames of amyloid-PET (ePET).

Subjects were divided into two groups: CU and CI. The CU included subjects without cognitive impairment (worried well and subjective cognitive decline), while CI included subjects with MCI or dementia defined based on clinical diagnostic criteria [13, 14].

The study protocol, including imaging studies, was approved by the local ethics committee (CCER numbers: 2020–00403 and PB_2016 - 01346), and the research was conducted in accordance with the Declaration of Helsinki and International Conference on Harmonization Good Clinical Practice guidelines. All subjects or their relatives provided voluntary written informed consent.

### Imaging protocol

#### MRI

Brain scans were acquired in the Division of Radiology of the Geneva University Hospitals using a 3 T MRI scanner (Magnetom Skyra, Siemens Healthcare, Erlangen, Germany) with 64-channel head coil. The MRI protocol included a structural T1-weighted 3D sequence (repetition time/echo time [TR/TE] = 1930/2.36 ms; flip angle = 8°; resolution = 0.9 mm isotropic; 208 sagittal slices) and a pulsed ASL (pASL) sequence with flow-sensitive alternating inversion recovery (FAIR) labeling scheme (bolus duration/inversion time = 800/2000 ms, flip angle = 180°; in-plane resolution = 3 × 3 mm^2^, reconstructed resolution = 1.5 × 1.5 mm^2^ with interpolation; 40 slices; volumes = 4). This PASL sequence was of the 3D hybrid echo type, called “turbo gradient spin echo” (TGSE) by Siemens, corresponding to Philips’s GRASE scheme. A segmented acquisition of 12 segments was used. Turbo factor was 18, and EPI factor was 21. Square FOV size was 192 × 192 mm, and matrix size was 64 × 64. TE was 16.38 ms and TR 5000 ms.

#### Amyloid- PET

Amyloid-PET images were acquired using 18 F-Flutemetamol or 18 F-Florbetapir using a Siemens Biograph mCT or Biograph Vision PET-CT scanner (Siemens Medical Solutions, Hoffman Estates, IL, USA) following a previously described protocol [15]. Early-phase amyloid-PET (18 F-Florbetapir [eFBP] and 18 F-Flutemetamol [eFMM]) image acquisition began immediately after tracer injection (via venous cannula) [16, 17], with static images acquired for 5 min (eFBP) or 10 min (eFMM). Data were collected in a list mode and reconstructed using 3D OSEM 4 interactions 8 subsets and a 2-mm Gaussian filter at full width and half maximum (FWHM), resulting in images with 400 × 400 matrix with 1.01-mm isotropic voxels.

### Image processing and quantitative analysis

PASL data were analyzed using FSL 6.0.3 (part of FSL FMRIB, Oxford, UK) and BASIL toolbox (part of FSL). As per [18], BASIL assumes by default a single well-mixed tissue compartment model with no dispersion of the bolus of labeled blood water (https://asl-docs.readthedocs.io/en/latest/basilcmd.html). This model was used for quantifying CBF with default relaxation values (T1 = 1.3 s, T1b = 1.65 s). Equilibrium blood magnetization was estimated from the calibration image using CSF as reference region, and adaptive spatial regularization on perfusion was also applied. CBF maps were rigidly registered to the corresponding T1-weighted image (FLIRT) and spatially normalized to the 2-mm MNI space (FNIRT). To ensure robustness of ASL data, a visual quality assessment of pulsed ASL acquisition was performed by FBP (neuroradiologist with 17 years of experience in ASL reporting). Ninety-eight out of 144 acquisitions were excluded since the presence of artifacts (head motion, signal drop, geometric distortion, or macro-vascular bright spots) [19] and/or delayed arrival time of the cerebral flow did not allow the detection of the spin-marked signal at the level of the cerebral tissue [20]. While the former is represented by intra-arterial serpiginous pulsed ASL signal within basal cisterns and cortical sulci, the latter represents a combination of ATA and low pulsed ASL signal in arterial border zones. This ATA and border zone sign artifacts occur when the transit time of arterial blood from the labeling plane to the imaging plane is greater than the post-labeling delay (PLD, e.g., subjects with diminished cardiac output or arterial stenosis) [21]. In our study, the ASL signal intensity was normalized to the eight top slices of the cerebellum, leading to individual rCBF maps. We applied this restriction in order to consider only those slices with good quality and stable values, as incomplete coverage, artifacts, or reduced signal can be present in the lower cerebellar slices of ASL. Cerebellum has been widely used for normalization in perfusion-weighted MRI studies when considering cognitively impaired/Alzheimer’s disease individuals as this region should be preserved, though other areas as putamen [7] or pons [22] have been also used in previous studies comparing ASL and PET data. However, in this study, we decided to use the cerebellum and to further restrict it to the superior slices as they were well perfused in both ASL and PET, and they are supplied by superior cerebellar arteries (SCA), very close to posterior arteries and posterior communicating arteries of the Willis polygon. This can potentially limit the influence between anterior and posterior interconnections in normal conditions. It is also important to recognize that achieving efficient labeling is more difficult in the posterior circulation than in the anterior circulation, mainly because of their greater tortuosity in the neck, as often demonstrated in studies focusing on selective flow territory mapping with ASL [23, 24]. This is due in part to degenerative changes in the spine (such as uncovertebral hypertrophy and osteophytes), which occur during aging and indent the vertebral arteries, inducing alterations in their course, which become tortuous and elongated. This is even more challenging when using pCASL schemes than PASL, as the spatial labeling location is very important for the labeling efficiency and should be thus carefully considered in similar studies. PET images were processed using in-house MATLAB code and SPM12 software package (Wellcome Department of Cognitive Neurology, London, UK) as described in a previous study [15]. Both pulsed ASL and ePET were processed in the same MNI space. ePET images in the MNI space were intensity normalized using the same reference region as for pulsed ASL, resulting into standardized uptake value ratio (SUVr) maps.

For each subject, we restricted the analysis of both maps to 17 anatomical regions selected from the Automated Anatomical Labeling Atlas (AAL3, Supplementary Fig. 1): 11 cortical areas (superior frontal, superior parietal, posterior cingulate, precuneus, superior temporal pole, superior temporal, inferior temporal, middle temporal, insula, angular gyrus, calcarine) and 6 subcortical areas (amygdala, hippocampus, caudate, pallidum, putamen, thalamus). Moreover, we extracted a composite mask including typical AD hypometabolic regions (metaROI) [25, 26].

### Statistical analysis

Demographics and clinical information are reported as mean ± standard deviation. We considered outliers of the rCBF values if the values were above or below two standard deviations from the mean and consequently removed the brain region. To evaluate the association between rCBF and SUVr, we performed multiple linear regression models between ASL and ePET measurements corrected by age across the 18 brain regions, correcting for multiple comparisons using the Bonferroni-Holm (BH) method. In addition, to compare the replicability of both techniques, we have calculated the within-subject coefficient of variation (wsCV) for each region. To guarantee that both ASL and ePET measurements have the same range in the wsCV calculation and Bland–Altman plots, they were standardized according to the respective mean. The wsCV was calculated by first determining the standard deviation of the difference between rCBF and SUVr for each subject and then dividing this by the mean of those differences. The resulting value was multiplied by 100 to express the wsCV as a percentage, indicating the relative variability between rCBF and SUVr. The differences between CU and CI in both rCBF and SUVr in the aforementioned 18 regions were tested using linear regression models adjusted by age. We set the alpha value for 5%; thus, we considered statistically significant results if *p* < 0.05.

## Results

### Participants

Our final sample was composed of a total of 46 subjects: 17 CU, 29 CI (25 MCI and 4 dementia). The characteristics of our cohort are reported in Table 1.

**Table 1:** Main demographic, neuropsychological and imaging features of the study cohort

Variables	Geneva Memory Center *N* = 46
Age, years	69 ± 8
Gender, female, *N* (%)	37 (80%)
Education, years	14 ± 4
MMSE	27 ± 3
Diagnosis, *N* (%)	
Cognitively unimpaired	17 (37%)
MCI	25 (54%)
Dementia	4 (9%)
Amyloid PET, positivity, *N* (%)	21 (46%)
Amyloid PET tracer, *N* (%)	
eFBP	24 (52%)
eFMM	22 (48%)

Numerical data are reported as mean ± standard deviation. Categorical data are reported as number (%). *MMSE*, Mini-Mental State Examination; *MCI*, mild cognitive impairment; *PET*, positron emission tomography.

Amyloid PET positivity has been defined based on visual assessment of an expert (> 15 years) nuclear medicine physician (VG, according to the standard procedure: https://www.ema.europa.eu/documents/product‐information/vizamyl‐epar‐product information_en.pdf; https://www.ema.europa.eu/documents/product‐information/amyvid‐epar‐product information_en.pdf).

### Linear regressions and wsCV between rCBF and SUVr

**Fig. 1:**
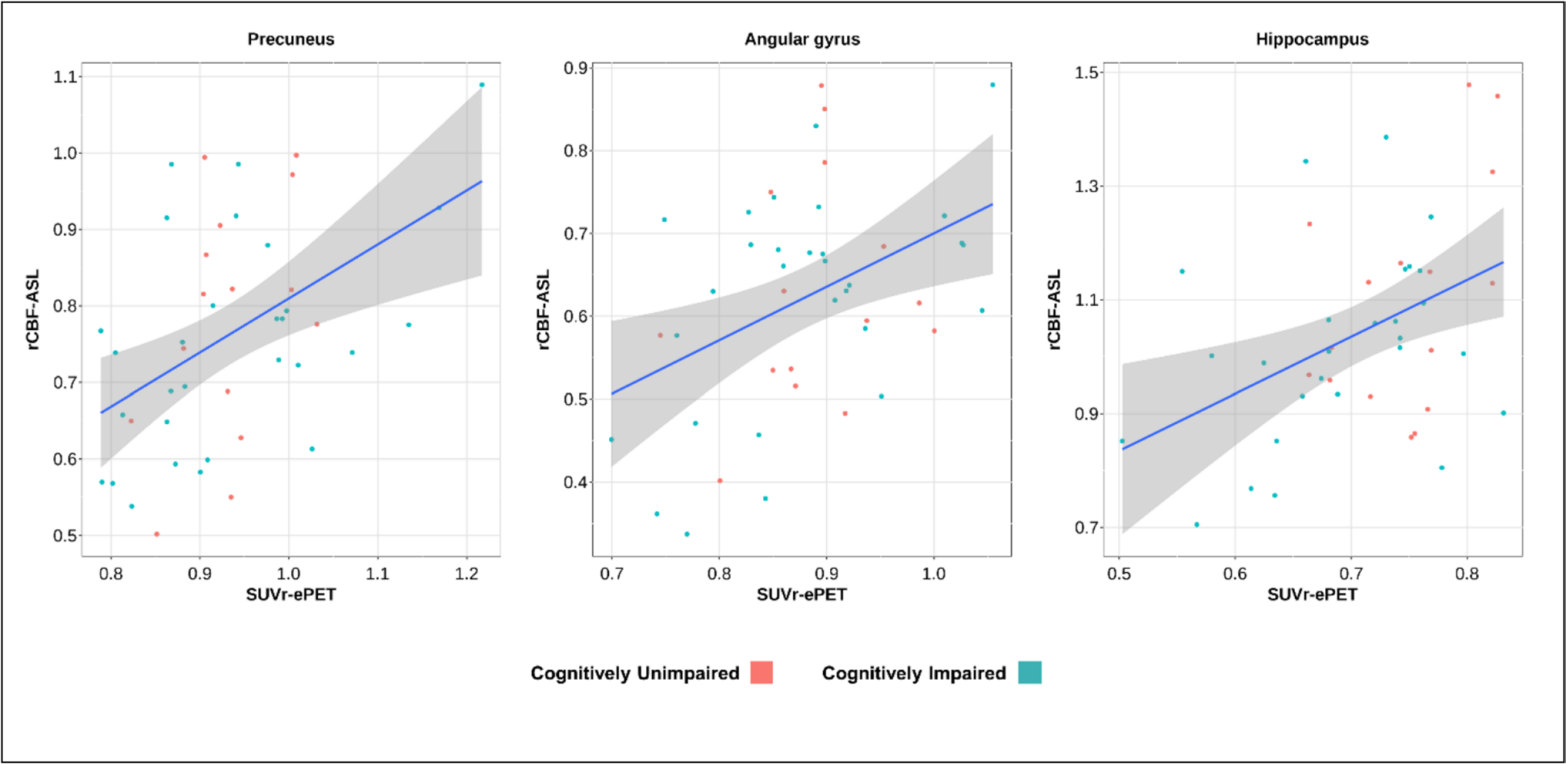
Representative scatterplots of the significant associations between rCBF and SUVr values. Blue lines show the linear regression between both techniques, and gray areas represent 95% confidence intervals

**Fig. 2:**
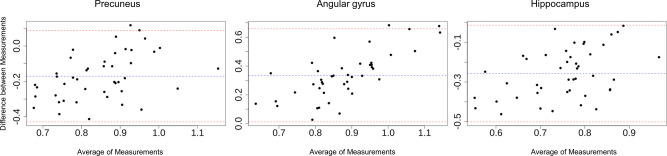
Bland–Altman plots comparing rCBF and SUVr values in regions with significant correlations. The plots display the differences between the two techniques (*y*-axis) against their average (*x*-axis) for each subject. The blue dashed line represents the mean difference (bias), while the red dashed lines indicate the upper and lower limits of agreement (mean difference ± 1.96 times the standard deviation of the differences)

In cortical regions, we only observed significant association between rCBF and SUVr in the precuneus (*β* = 0.69; *p* = 0.01), and angular gyrus (*β* = 0.64; *p* = 0.04) after the BH multiple comparison correction (Table 2; Fig. 1). Likewise, in subcortical regions, rCBF and SUVr were only significantly correlated in hippocampus (*β* = 1.23, *p* = 0.01) (Table 2; Fig. 1). The wsCV in the aforementioned regions was lower compared to others (wsCV_precuneus_ = 16.9%; wsCV_angular_ = 19.4%; wsCV_hippocampus_ = 16.1%) (Fig. 2), although low variability was also observed in calcarine (wsCV = 16.3%) and thalamus (wsCV = 17.4%) (Table 2).

**Table 2:** Linear regression and wsCV between rCBF and SUVr and regional differences between cognitively unimpaired (CU) and cognitively impaired (CI)

	Correlation and wsCV between rCBF and SUVr				Modality	Mean ± standard deviation		CU vs CI
	*β*	*p*-value	wsCV	*n*		CU	CI	*p*-value
Cortical ROIs								
Frontal superior	0.25	0.33	27.5	45	ePET	0.83 ± 0.07	0.84 ± 0.12	0.89
					pASL	0.43 ± 0.1	0.44 ± 0.14	0.39
Parietal superior	0.19	0.33	30.6	44	ePET	0.69 ± 0.12	0.70 ± 0.12	0.71
					pASL	0.33 ± 0.09	0.33 ± 0.1	0.88
Posterior cingulate	0.25	0.57	20.5	44	ePET	0.91 ± 0.07	0.82 ± 0.13	0.059
					pASL	1.06 ± 0.18	0.92 ± 0.13	**0.003**
Precuneus	0.69	**0.01**	16.9	44	ePET	0.93 ± 0.06	0.93 ± 0.11	0.93
					pASL	0.81 ± 0.14	0.74 ± 0.15	**0.034**
Superior temporal pole	0.36	0.66	32.1	44	ePET	0.59 ± 0.05	0.58 ± 0.06	0.87
					pASL	0.69 ± 0.21	0.61 ± 0.19	0.11
Superior temporal	0.40	0.65	23	43	ePET	0.91 ± 0.05	0.89 ± 0.08	0.72
					pASL	0.86 ± 0.21	0.87 ± 0.19	0.66
Inferior temporal	0.04	0.93	26.9	44	ePET	0.88 ± 0.05	0.88 ± 0.12	0.65
					pASL	0.49 ± 0.13	0.42 ± 0.09	0.089
Middle temporal	0.19	0.66	21.1	43	ePET	0.91 ± 0.05	0.89 ± 0.08	0.69
					pASL	0.67 ± 0.17	0.65 ± 0.12	0.71
Insula	0.34	0.66	26.2	43	ePET	0.94 ± 0.07	0.89 ± 0.09	0.46
					pASL	0.96 ± 0.26	0.91 ± 0.21	0.26
Angular gyrus	0.64	**0.04**	19.4	44	ePET	0.88 ± 0.07	0.88 ± 0.10	0.64
					pASL	0.63 ± 0.14	0.62 ± 0.13	0.7
Calcarine	− 0.04	0.94	16.3	44	ePET	1.05 ± 0.06	1.06 ± 0.09	0.55
					pASL	1.18 ± 0.11	1.04 ± 0.16	**0.01**
Subcortical ROIs								
Amygdala	0.51	0.66	29.5	44	ePET	0.74 ± 0.04	0.73 ± 0.07	0.66
					pASL	0.87 ± 0.24	0.80 ± 0.24	0.14
Hippocampus	**1.23**	**0.01**	16.1	43	ePET	0.75 ± 0.05	0.69 ± 0.08	**0.039**
					pASL	1.13 ± 0.21	1.00 ± 0.15	**0.016**
Caudate	0.33	0.12	28.1	44	ePET	0.64 ± 0.10	0.58 ± 0.14	0.38
					pASL	0.55 ± 0.13	0.50 ± 0.12	0.15
Pallidum	− 0.02	0.96	27.2	45	ePET	1.06 ± 0.07	1.05 ± 0.09	0.89
					pASL	0.84 ± 0.23	0.84 ± 0.21	0.77
Putamen	0.07	0.93	23.2	44	ePET	1.13 ± 0.06	1.16 ± 0.09	0.2
					pASL	0.85 ± 0.19	0.81 ± 0.18	0.4
Thalamus	0.64	0.12	17.4	46	ePET	1.13 ± 0.07	1.11 ± 0.11	0.77
					pASL	1.14 ± 0.21	1.07 ± 0.19	0.17
metaROI								
Composite	0.33	0.57	22.3	44	ePET	0.98 ± 0.08	0.96 ± 0.1	0.82
					pASL	0.92 ± 0.21	0.8 ± 0.16	**0.034**

Pearson correlation coefficients and corresponding *p*-values are reported for each cortical and subcortical region of interest (ROIs) from the AAL3 atlas as well as for the MetaROI. Significant *p*-values are reported in bold.

### Differences in rCBF and SUVr between CU and CI

**Fig. 3:**
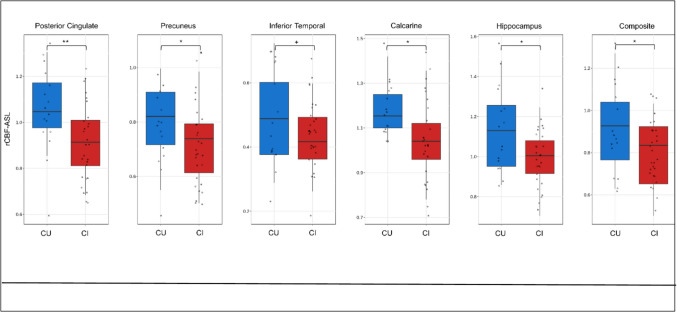
Boxplots showing medians and interquartile ranges of rCBF for the regions with significant between-group differences according to the linear regression model. Asterisks represent significant results. + *p* < 0.1; *p < 0.05; **p < 0.01

**Fig. 4:**
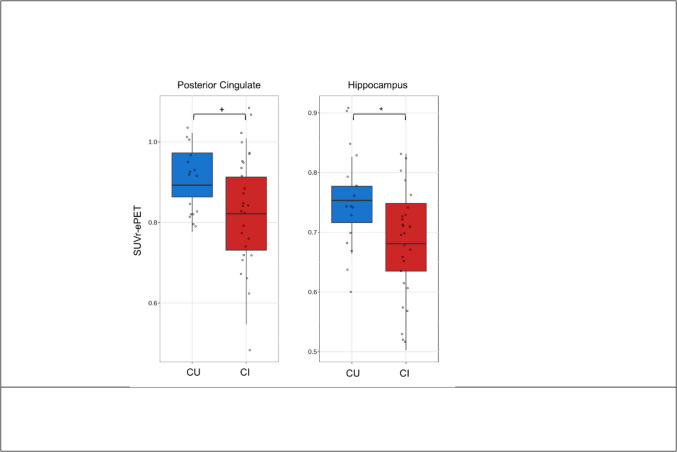
Boxplots showing medians and interquartile ranges of SUVr for the regions with significant between-group differences according to the linear regression model. Asterisks represent significant results. + *p* < 0.1; *p < 0.05

**Fig. 5:**
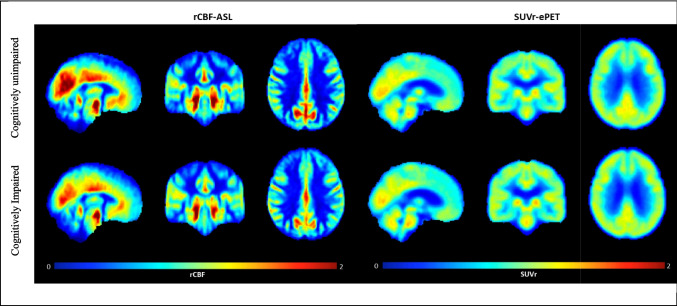
Average rCBF-pASL and SUVr-ePET maps for the two groups (CU, top; CI, bottom). All of the slices represented following radiological convention, and the same range (0–2, dimensionless) was used for both modalities

We found statistically significant differences in rCBF between CU and CI in several regions, including posterior cingulate, precuneus, hippocampus, calcarine, and composite region (*p* < 0.05; Table 2). In addition, we observed a marginally significant difference in the inferior temporal region (*p* = 0.089) (Fig. 3). Regarding SUVr, significant differences were observed between the two groups in the hippocampus (*p* < 0.05; Table 2), with marginally significant differences in posterior cingulate (*p* = 0.059) (Fig. 4). All other comparisons were not statistically significant (*p* > 0.05; Table 2). The rCBF and SUVr maps for CU and CI showed consistent spatial patterns as judged by visual inspection by an expert neuroradiologist (FBP > 15 years), although the contrast between cortical gray and white matter was more pronounced in pulsed ASL than ePET (Fig. 5). Moreover, we acknowledge that the smoothness of the ASL maps was not equalized to that of PET, and this could partially limit the comparison in small ROIs, as the hippocampus. However, since our main objective is to adhere to a clinical scenario, further smoothing of the ASL maps reported in Fig. 5 has been avoided, which would have made it less clinically useful. ASL also seems to have higher sensitivity to hyperintense vascular signal with a residual vascular component still present, even if clinically acceptable based on the visual quality assessment.

## Discussion

Our findings reveal significant associations between rCBF and SUVr in regions such as the precuneus, hippocampus, and angular gyrus. The clinical significance of these findings aligns with previous studies that demonstrated reduction in glucose metabolism in the precuneus and hippocampus in individuals with cognitive impairment due to AD [27, 28].

While our study observed similar associations in the frontal and temporal regions between pulsed ASL and ePET, compared to a previous study involving individuals at risk for autosomal AD [10], we found stronger association between both measures in the hippocampus, as well as a very similar association in the parietal region. Notably, the previous study did not test the association between ASL and ePET in angular gyrus. Methodological differences between the previous study and our current research must be considered. For instance, our sample was drawn from a heterogeneous memory clinic cohort, while the previous study exclusively included individuals at risk for autosomal AD. Moreover, differences between the ePET protocols and the radiotracers used in each study (static vs dynamic and eFBP/eFMM vs PIB) could influence perfusion results. Different perfusion proxies can be calculated using PET imaging: while estimates of the delivery rate derived from dynamic images (R1) are more precise but also more complex to be derived, regional uptake values calculated on static early frame images provide a simpler and more easily accessible measure. Parameters that differ across studies are thus the dynamic vs. static nature of the PET acquisition, the duration of the static acquisition, and the tracer used, as the tracer lipophilicity and related extraction fraction influence the correlation of these parameters with cerebral blood flow [29]. Indeed, we used eFBP and eFMM tracers with static images because we have recently demonstrated their capability to detect perfusion with a similar efficacy to FDG-PET [30]. On the other hand, early PIB-PET uptake shows slightly lower association than the ratio of tracer influx extracted from dynamic PIB-PET in comparison to FDG-PET [31], as well as different spatial patterns between both techniques [10, 32]. Likewise, although pulsed ASL has shown similar topographic patterns in comparison with FDG-PET [10], significant differences exist between both techniques, mostly in subcortical areas [33]. Interestingly, our maps revealed substantial overlap between pulsed ASL and ePET, despite the greater dynamic range visually observed with pulsed ASL. The disparity in results could potentially be attributed to the high variance in ASL analysis due to a lower signal-to-noise ratio when compared to the ePET, as previously reported [11].

Our findings also indicated significant differences between CU and CI in rCBF in the posterior cingulate, with SUVr showing marginally significant differences in this region. This is partly consistent with a previous study that observed significantly different CBF-ASL in the posterior cingulate between subjects with AD and controls, while no differences were detected using ePET [11]. Pulsed ASL was also able to differentiate CU and CI through CBF levels in the precuneus, hippocampus, calcarine, and composite region (*p* < 0.05) and inferior temporal (*p* < 0.1). While differences in precuneus, hippocampus, and inferior temporal regions were expected given the course of neurodegeneration in AD [34], the findings concerning the calcarine were unexpected, as this region is typically more affected by aging rather than AD [35]. Nonetheless, the effect persisted even after adjusting for age. Likewise, our study found that CBF-ASL and ePET had the ability to differentiate cognitive status when measured in hippocampus. The hippocampus is of particular interest considering the topographical course of neurodegeneration in AD. In general, the results of this study suggest that both approaches successfully detected neurodegeneration in brain regions associated with CI due to AD. However, rCBF showed superior performance in cortical areas, while SUVr was more effective in discriminating groups only in hippocampus. Indeed, rCBF already had been shown to provide negative associations in subcortical regions when compared to FDG-PET, perhaps indicating the existence of neurovascular decoupling along the AD continuum [10].

The observed differences between PASL and ePET likely stem from the distinct physiological processes each technique measures. ePET provides an estimate of tracer delivery, which reflects relative tissular cerebral blood flow and is closely related to regional brain function and metabolism. Because ePET integrates signal over a longer acquisition time (5–10 min), it is less affected by transit time variations or vascular effects and has shown strong correlations with FDG-PET in previous studies [30]. In contrast, PASL directly quantifies cerebral blood flow using an endogenous tracer. However, PASL is more sensitive to variations in arterial transit time and vascular components, particularly in regions with delayed flow, such as the posterior circulation or border zones. These methodological differences may explain why rCBF-ASL detected more significant differences between CU and CI groups than SUVr-ePET. The higher sensitivity of PASL to vascular factors may enhance its ability to capture perfusion alterations in the early stages of cognitive impairment, while ePET, due to its metabolic correlation, may be less sensitive to these changes.

Differences observed between pulsed ASL and ePET could potentially be attributed to variations between both methods when compared to FDG-PET [7–9, 30, 31]. In fact, the Bland–Altman plots (Fig. 2) suggest a proportional bias between ASL and ePET given that the difference between the two measurements changes with the increase of their average magnitude. This may be explained by the distinct physiological processes measured by ASL and ePET, which might not correlate linearly, resulting in increased disparities at higher values. Therefore, future research should focus on investigating a comparative analysis of CBF-pASL and SUVr-ePET in relation to FDG-PET to better understand their respective associations with the established measure of hypometabolism in patients with suspected neurodegeneration.

Another hypothesis is that amyloid deposition might impact differently the perfusion assessment by pulsed ASL and ePET. For instance, a previous study observed different agreement between ePIB-PET and FDG-PET according to the amyloid status [31]. However, a recent study showed high agreement between ePET (using eFBP and eFMM tracers) and FDG-PET regardless of the amyloid positivity of the subjects [30]. Similarly, previous studies have shown an inverse relationship between amyloid deposition and CBF-ASL/FDG-PET [10]. It is important to note that the strength of these correlations varied between the two imaging techniques. Specifically, rCBF was found to be mostly reduced in cortical areas, while glucose metabolism exhibited greater reductions in subcortical regions [10]. Hence, perfusion assessment conducted using pulsed ASL or ePET may vary based on the distribution of amyloid deposits in cortical and subcortical areas throughout the AD spectrum.

## Limitations and future directions

The primary limitation of this study is its retrospective nature. Moreover, one of the main limitations is the relatively small sample size. Additionally, our study lacks a comparison with FDG-PET, the standard measure of metabolism.

We acknowledge that our ASL data are acquired over a decade ago and that FAIR labeling is no longer considered the gold standard for the pulsed ASL. However, at the time of data collection (2016–2021), FAIR-based PASL was widely used, and studies have demonstrated good reliability of FAIR labeling in perfusion quantification [36]. The ASL White Paper [37] recommended the use of pCASL, which has since become the preferred method due to its higher signal-to-noise ratio (SNR) and improved robustness across clinical conditions [38]. While several studies have compared pCASL with PET techniques—such as [39] pCASL vs. ^15^O-H₂O PET) and [10] (pCASL vs. FDG-PET and PIB-PET)—to our knowledge, no studies have systematically evaluated PASL in comparison to early-phase amyloid PET (ePET) in a memory clinic cohort. Given that PASL remains the only available ASL option in certain clinical centers, our study provides valuable insights into its agreement with ePET in a memory clinic population. However, we recommend that future studies preferably employ pCASL and explore multi-time point ASL acquisitions for further comparisons with ePET.

Furthermore, the intrinsic resolution of the two techniques is very different, and no smoothing has been applied to equalize them. Future studies should test the impact of the resolution on the association between ePET and ASL.

Lastly, given that we intended to compare the two techniques in a clinical setting, we did not correct for partial volume effect (PVE). This is particularly relevant because PVE may lead to biased results in areas with atrophy, which are important for understanding AD pathology [34]. Therefore, although previous studies have shown consistent findings in the perfusion patterns of patients with AD without PVE correction [40], pulsed ASL and ePET might be impacted differently. Moreover, future studies aimed at improving the comparison between the two techniques even outside clinical settings should consider equalizing their smoothness, if PVE is not performed. Indeed, depending on smoothness and effective spatial resolution, signal contributions from neighboring regions may affect a specific ROI, particularly for ROIs as small as the hippocampus, and thus should be taken into account. Finally, we acknowledge that the effective spatial resolution and PVE are also relevant for the accurate GM and WM CBF contrast assessments with ASL.

## Conclusion

Taken together, these findings might suggest that perfusion levels measured in pulsed ASL or in PET might behave differently in different brain regions. Both techniques were sensitive to perfusion in different regions, especially with ASL showing more difference in CU vs CI. While this study does not assess the direct clinical implementation of ASL or ePET, it serves as an important first step in understanding their comparability. Future studies should focus on evaluating the clinical utility of these measures, including direct comparisons with FDG-PET, larger and more diverse samples, and longitudinal analyses across different disease stages to determine their potential role in clinical workflows.

## Supplementary Information

Below is the link to the electronic supplementary material.

Supplementary file1 (DOCX 340 KB)

## Data Availability

Data are available upon reasonable request to the PI (Prof. Giovanni B. Frisoni).
