# Supplementary material for: Agreement between early-phase amyloid-PET and pulsed arterial spin labeling in a memory clinic cohort
Source: J Mol Med (Berl). 2025 May 20;103(7):809–19. doi: 10.1007/s00109-025-02545-w (PMC12287194; doi:10.1007/s00109-025-02545-w)
Supplement: Supplementary file 1 — Supplementary file1 (DOCX 340 KB) [file 109_2025_2545_MOESM1_ESM.docx]

Supplementary material

**Supplementary Figure 1.** Regions of interest


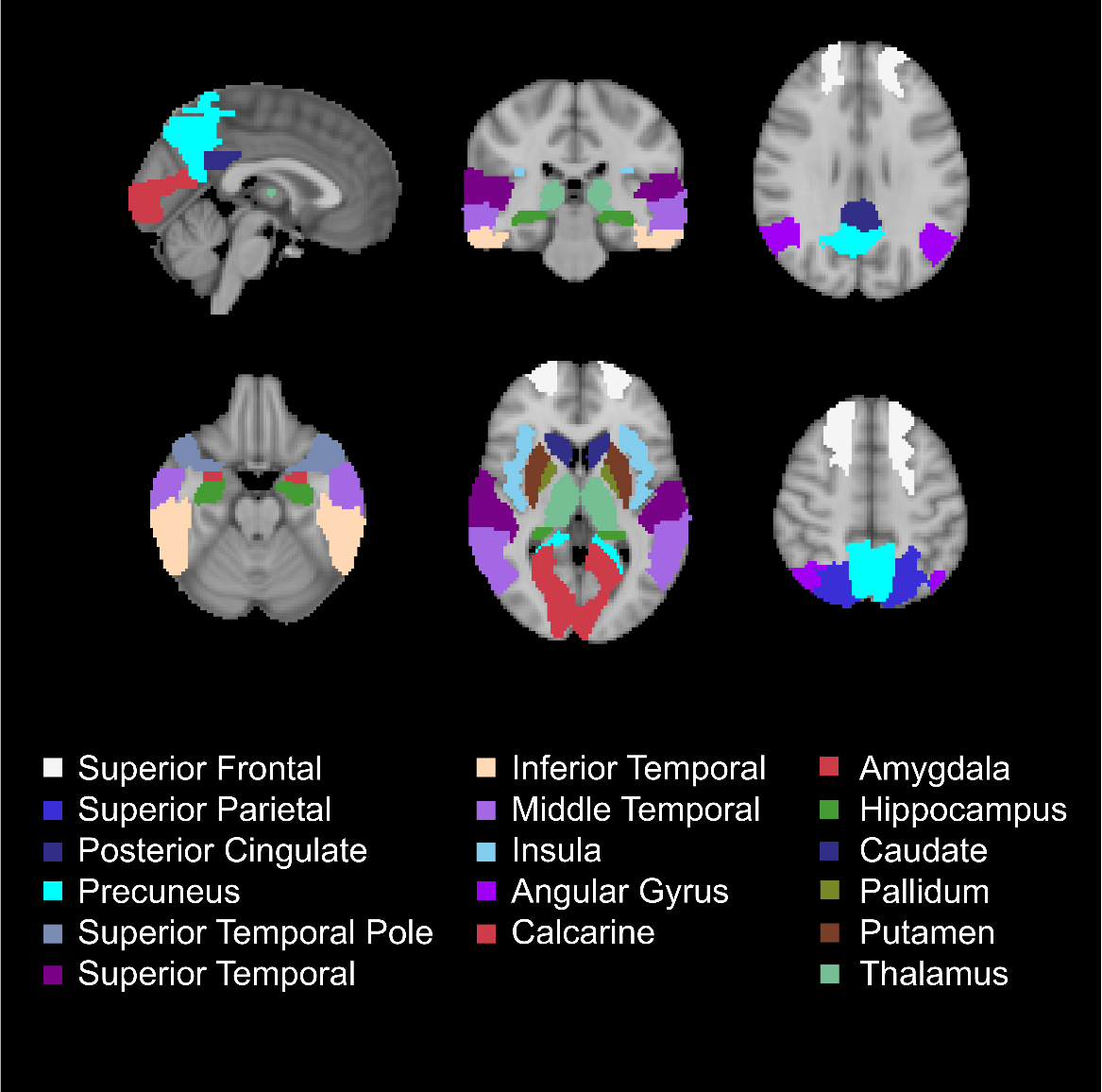


The figure shows the 17 anatomical regions selected from the Automated Anatomical Labeling Atlas that were chosen for the statistical analysis: 11 cortical areas (superior frontal, superior parietal, posterior cingulate, precuneus, superior temporal pole, superior temporal, inferior temporal, middle temporal, insula, angular gyrus, calcarine), and 6 subcortical areas (amygdala, hippocampus, caudate, pallidum, putamen, thalamus).
